# Effect of etelcalcetide versus alfacalcidol on left ventricular function and feature-tracking cardiac magnetic resonance imaging in hemodialysis—a post-hoc analysis of a randomized, controlled trial

**DOI:** 10.1186/s12968-023-00975-4

**Published:** 2023-11-06

**Authors:** Katharina Dörr, Andreas Kammerlander, Francesco Lauriero, Matthias Lorenz, Rodrig Marculescu, Dietrich Beitzke

**Affiliations:** 1 Department of Nephrology, Medical University of Vienna, Vienna, Austria; 2 Department of Cardiology, Medical University of Vienna, Währinger Gürtel 18-20, 1090 Vienna, Austria; 3 Department of Radiological and Hematological Science, Section of Radiology, Università Cattolica del Sacro Cuore, Rome, Italy; 4 Vienna Dialysis Center, Vienna, Austria; 5 Department of Laboratory Medicine, Medical University of Vienna, Vienna, Austria; 6 Department of Biomedical Imaging and Image-Guided Therapy, Division of Cardiovascular and Interventional Radiology, Medical University of Vienna, Vienna, Austria

**Keywords:** Etelcalcetide, Left ventricular function, Hemodialysis, Cardiac magnetic resonance imaging, Global longitudinal strain

## Abstract

**Background:**

Calcimimetic therapy with etelcalcetide (ETEL) has been shown to attenuate the advancement of left ventricular (LV) hypertrophy in hemodialysis patients measured by cardiac magnetic resonance (CMR). The aim of the study was to evaluate whether this effect is accompanied by alterations in LV function and myocardial composition.

**Methods:**

This was a post-hoc analysis of a randomized-controlled trial of ETEL versus Alfacalcidol (ALFA) in 62 hemodialysis patients. LV function was assessed using LV ejection fraction (LVEF) and LV global longitudinal strain (GLS) on feature-tracking (FT) CMR. Myocardial tissue characteristics were analyzed using parametric T1 and T2 mapping.

**Results:**

Of the total study cohort (n = 62), 48 subjects completed both CMR scans with sufficient quality for FT analysis. In the one-year follow-up, LV GLS deteriorated in the ALFA group, whereas the ETEL group remained stable (LV GLS change: + 2.6 ± 4.6 versus + 0.3 ± 3.8; p = 0.045 when adjusting for randomization factors and baseline LV GLS). We did not observe a difference in the change of LVEF between the two groups (p = 0.513). The impact of ETEL treatment on LV GLS over time remained significant after additional adjustment for the change in LV mass during the study period. ETEL treatment did not significantly affect other CMR parameters. There were no changes in myocardial composition between treatment groups (T1 time change: + 15 ± 42 versus + 10 ± 50; p = 0.411; T2 time change: − 0.13 ± 2.45 versus − 0.70 ± 2.43; p = 0.652).

**Conclusions:**

In patients undergoing hemodialysis, treatment with ETEL was protective against deterioration of LV longitudinal function, as evaluated through FT CMR, when compared to the control therapy of ALFA. This effect was not mediated by the change in LV mass.

*Trial registration* URL: https://clinicaltrials.gov/ct2/show/NCT03182699. Unique identifier: NCT03182699.

**Supplementary Information:**

The online version contains supplementary material available at 10.1186/s12968-023-00975-4.

## Introduction

Chronic kidney disease (CKD) is associated with an increased risk of cardiovascular morbidity and mortality [1, 2]. It results from a combination of both traditional and kidney specific risk factors, which increase as CKD advances and heart failure (HF) is common at every stage [3]. Global longitudinal strain (GLS), which reflects the longitudinal contraction of the myocardium, is an alternative approach to assess left ventricular (LV) systolic function in addition to LV ejection fraction (EF) and is a measure of subclinical systolic dysfunction [4]. It predicts prognosis across a broad spectrum of patients with HF, including HF with preserved EF which is known to be highly prevalent in hemodialysis patients. It results from the effects of pre-existing comorbidities, the continuous strain put on the myocardium through hemodialysis treatment and ultrafiltration as well as the effects of chronic fluid overload [5–7]. GLS is reported as a negative value in percentages, and a value closer to zero is a sign of impaired LV function, which is associated with an increased risk of mortality in both pre-dialysis and dialysis patients [8].

We previously demonstrated, in a randomized controlled trial of hemodialysis patients with secondary hyperparathyroidism that treatment with the calcimimetic etelcalcetide (ETEL) could inhibit the progression of LV mass index compared to active vitamin D therapy with alfacalcidol (ALFA) and that this was associated with the drug-induced suppression of fibroblast growth factor 23 (FGF23) [9]. Declining kidney function leads to alterations in the vitamin D metabolism, i.e. lower levels of calcitriol lead to a decrease in serum calcium and impaired phosphate excretion. Secondary hyperparathyroidism is an adaptive process which develops as a response to these dysregulations [10]. Low calcium, high phosphate and calcitriol deficiency lead to a higher synthesis of parathyroid hormone (PTH) and increased phosphatonin FGF23. The level of FGF23 in CKD patients is an independent risk factor for a progression to end-stage renal disease (ESRD) and is associated with cardiovascular- and all-cause mortality [11]. Both vitamin D and calcimimetics such as cinacalcet or ETEL are commonly used treatment options for secondary hyperparathyroidism [12]. Vitamin D treatment aims at compensating the calcitriol deficiency and elevates the intestinal reabsorption of calcium and phosphate, thereby suppressing the synthesis of PTH [13]. Calcimimetics interact with the calcium sensing receptor in the parathyroid gland, increasing its sensitivity for circulating calcium and subsequently lowering the concentrations of PTH and FGF23 [14].

Currently, there is little data available regarding the impact of calcimimetics on cardiac function and fibrosis in humans [15, 16]. In a large non-CKD cohort, an independent association was observed between FGF23 levels and increased LV mass and lower LV systolic function [17].

Based on our previous findings indicating the inhibition of LV hypertrophy (LVH) progression with ETEL, we have developed the hypothesis that this intervention may also lead to alterations in LV function and fibrosis.

Cardiac magnetic resonance (CMR) was used to measure cardiac function, including GLS through feature-tracking (FT), as well as to examine myocardial tissue characteristics using parametric mapping techniques [18–20].

## Methods

### Study design and participants

This randomized, controlled, single-blinded study ETECAR-HD (Effect of etelcalcetide on cardiac hypertrophy in hemodialysis patients) involved a cohort of 62 patients undergoing maintenance hemodialysis who had secondary hyperparathyroidism (i.e. PTH levels ≥ 300 ng/l) and pre-existing LVH, defined as a septum thickness of a minimum of 12mm in the screening echocardiography [21]. Patients with an unstable medical condition and significantly impaired LV systolic function or symptomatic severe heart valve defects were excluded from the trial [22, 23].

Enrollment for the trial was conducted at two dialysis centers located in Vienna, Austria. Participants were deemed eligible only if they exhibited stable volume status.

Further information regarding the study design, as well as the entire list of inclusion and exclusion criteria, have been previously reported [9, 24]. Approval for the trial was obtained from the ethics committee of the Medical University of Vienna (EK # 1127/2017) as well as the national regulatory authorities (AGES # 10087746) and conducted in adherence to the principles outlined in the Declaration of Helsinki.

### Interventions and measurements

Patients were randomized to receive either intravenous ETEL or ALFA medication for a period of one year. The treatment initiation involved a dosage of 5 mg or 1μg three times per week after hemodialysis, which was adjusted every four weeks to achieve a target PTH-value of 100-300ng/l while considering of calcium and phosphate levels (2.08–2.55 mmol/l and ≤ 2.5 mmol/l respectively). To avoid confounding of the treatment effect by volume overload, body composition monitoring was conducted. It was performed during the screening phase, followed by regular measurements every two months throughout the duration of the trial [25]. In cases of hypervolemia, adjustments to the dry weight were made based on clinical judgement and standard of care.

### Study outcomes

The objective of this analysis was to investigate whether the use of ETL compared to ALFA medication was linked to alterations in systolic dysfunction and cardiac fibrosis, in addition to the previously reported effect on the progression of LVH [9].

### Cardiac imaging

All CMR examinations were performed on a mid-week non-dialysis day, using a 1.5 Tesla scanner (Siemens Avanto FIT 1.5 T, Siemens Healthineers, Erlangen Germany) at baseline and after one year of treatment. For evaluation of cardiac mass, steady-state free precession imaging was acquired in two-, three-, and four-chamber views and short axis slices. Short axis was covered by eleven slices (slice thickness: 0.6.0 mm) with a distance factor of 58%. Repetition time (TR) was 52.2 ms and echo time (TE) was 1.21 ms.

T1-mapping was performed using an electrocardiographically triggered modified Look Locker inversion recovery (MOLLI) sequence based on a 5[3]3 prototype (five acquisition heartbeats are followed by three recovery heartbeats and a further three acquisition heartbeats) on three short-axis slices (basal/mid-cavity and apical), including inline motion correction. T1 sequence parameters were: starting inversion time (TI) = 120 ms; TI increment = 80 ms; flip angle 35°; reconstructed matrix size = 256 × 218; and acquired matrix size = 256 × 144 (phase encoding resolution = 66%, phase encoding field of view = 85%).

For T2 -mapping a T2 prepared sequence with balanced steady-state free precession readout was used (TR/TE: 193.27/1.07 ms; flip angle 70°; resolution 1.9 × 1.9 mm) [26]. T2 mapping was performed with the same short axis slices as T1-mapping.

To quantify LV function and mass, feature tracking, as well as mapping parameters commercially available cardiac postprocessing software was used (QMass/QMap,QStrain, Medis Medical Imaging 4.0; Leiden, The Netherlands). LV mass index was obtained by normalizing LV mass to body surface area [27]. T1 and T2 maps were analyzed segment-wise based on the AHA 16-segment model and averaged for statistical analysis.

**Fig. 1:**
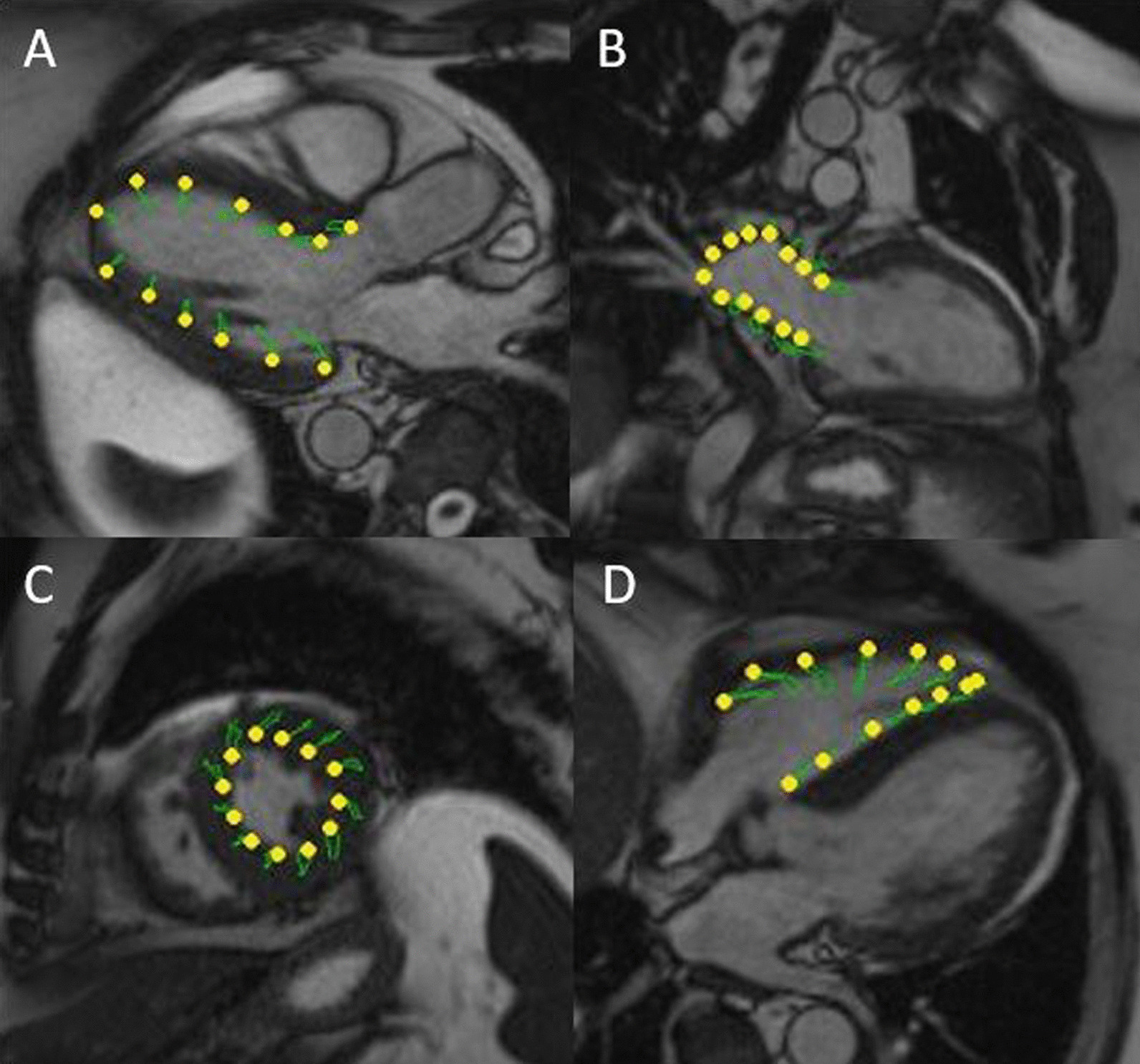
GLS and GCS measurement in CMR. End systolic and end diastolic contouring of long axis (**A**, **B**, **D**) and short axis (**C**) cine images using a feature tracking software. GLS and GCS is derived from long axis cine (**A**), RV Strain parameters are derived from four chamber cine (**B**), GCS is derived from short axis cine (**C**). LA strain parameter are derived from two chamber cine images (**D**)

Feature tracking parameters were analysed using standard two-, three-, and four chamber cine views for global longitudinal strain and atrial strain parameters, as well as short axis cine for global radial and global circumferential strain parameters. For the assessment of global longitudinal strain (GLS) and global circumferential strain (GCS), long axis and short axis cine SSFP were analysed using a feature-tracking software (QStrain, Medis Medical Imaging, Leiden, NL). Long axis cine images in standard standard two-, three, and four chamber view as well as short axis slices were semiautomatically contoured at the endocardium in systole and diastole and were afterwards manually corrected before data extraction (see Fig. 1). The long axis two chamber view was used for the evaluation of the left atrial strain. Contours of the left atrium were drawn manually in end systole as well as end diastole. For the evaluation of RV strain long axis four chamber view was used and contouring of the RV borders was done in end systole as well as end diastole. All contouring was performed by a cardiac imaging specialist with more than ten years of experience in CMR who was blinded for the treatment group according to the recommendations of the Society of Cardiac Magnetic Resonance (SCMR) [27].

### Laboratory measurements

Laboratory measurements were performed from blood samples collected at the beginning of dialysis sessions by the central laboratory of the Medical University of Vienna. N-terminal pro-brain natriuretic peptide (NT pro BNP) levels were assessed using an electro-chemiluminescent immunoassay (Cobas^®^, measurement in real time, coefficient of variation = 3.7%). Intact FGF23 levels were measured using a chemiluminescent immunoassay (DiaSorin^®^, measurement batched, duplicate, coefficient of variation ≤ 3.8%). These measurements were conducted at nine different timepoints throughout the trial.

### Sample size, randomization and blinding

This post-hoc analysis was based on a pre-calculated number of patients, which was originally determined to detect a between-group difference in the change of LV mass index [9]. Randomization was performed in permuted blocks of 4 stratified for dialysis center and residual kidney function (≥ 500 ml and < 500 ml urine per day). Both the participants and the radiologist responsible for assessing the CMR were unaware of the treatment allocation, ensuring a blinded approach.

### Statistical methods

We report mean and standard deviation for continuous parameters, and total numbers and percentages for categorical data. Comparisons between two groups were performed using the Wilcoxon rank sum test and chi-square test, as appropriate. We used the per-protocol dataset of the previously described cohort [9]. Normal distribution of metric variables was checked by the Shapiro–Wilk test and visual assessment.

The treatment effect on the change of CMR parameters was assessed using repeated measure analysis of covariance (ANCOVA) and multivariate linear regression models. In addition to the crude analysis, we adjusted the model for baseline LV GLS, male sex, LV mass change, and—in an exploratory step—FGF23 change.

Visual assessment of longitudinal data, stratified by treatment group, were displayed using the *marginsplot* command in Stata.

We used Stata 15.1 (StataCorp, College Station, Texas, USA) for all analyses and set the level of significance to an alpha of 0.05 unless stated otherwise.

## Results

### Participants

A total of 62 patients were recruited for the study and randomly assigned to receive either ETEL (n = 32) or ALFA (n = 30) therapy. Ten patients, five in each group, discontinued their participation in the trial. Reasons for drop-out were renal transplantation or discontinuation of hemodialysis due to renal recovery (n = 7); and death (n = 3). Patients who underwent renal transplantation during the trial were scheduled for a second CMR once they reached a clinically stable condition, which was after 1.8 months on average (between one to four months) after surgery.

No study medication was administered during this period. These patients received treatment for a duration of an average of 6.3 months (between three to nine months) prior to undergoing renal transplantation. The patient who discontinued hemodialysis was under study medication for 3.5 months before dropping out. He underwent his second CMR one year after his first and therefore did not receive study medication for 8.5 months. Due to the fact that patients who dropped out of the study but still underwent both baseline and follow-up CMR imaging had received study medication for approximately half of the originally planned study duration, and considering the average time gap between dropping out and the second CMR, the decision was made to use the per-protocol dataset. Out of the initial 62 patients, the remaining 52 individuals received the study medication for the entire duration of 12 months and underwent the second CMR immediately upon completion of the treatment.

**Fig. 2:**
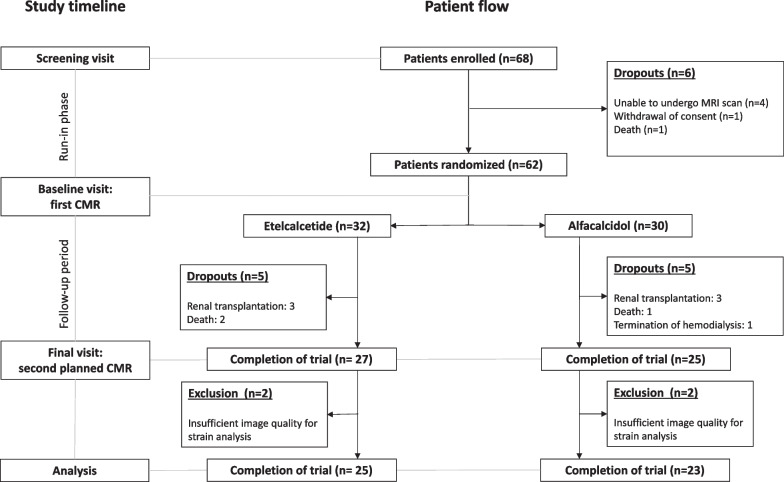
Consolidated Standards of Reporting Trials (CONSORT) flow diagram

Four patients were excluded due to insufficient imaging quality for accurate strain assessment. Among the excluded patients, three individuals experienced artifacts caused by arrhythmia (atrial fibrillation in two patients and multiple extrasystoles in one patient) while the fourth patient exhibited breathing motion artifacts on short axis images. Consequently, the cohort for the study consisted of 48 patients, Fig. 2.

Patient characteristics at baseline are reported in Table 1 and have been described previously [9, 28, 29]. The degree of overhydration, assessed through body composition monitoring at the screening stage and subsequently measured at two-month intervals throughout the trial, was found to be similar between the groups [9].

**Table 1:** Baseline characteristics of the study cohort. Per-protocol dataset with full strain assessment

	Total	ALFA	ETEL	p-value
	N = 48	N = 23	N = 25	
Age	62 (11)	61 (7)	62 (13)	0.88
Male sex	75%	83%	68%	0.24
Diabetes	46%	48%	44%	0.79
Hypertension	98%	96%	100%	0.29
Hyperlipidemia	52%	52%	52%	0.99
Coronary artery disease	58%	70%	48%	0.13
Heart failure	27%	22%	32%	0.42
Peripheral arterial disease	21%	22%	20%	0.88
Cause of ESRD				0.59
Diabetic nephropathy	27%	35%	20%	
Hypertensive nephropathy	15%	13%	16%	
Vascular nephropathy	19%	17%	20%	
Polycystic kidney disease	12%	17%	8%	
Glomerulonephritis	10%	9%	12%	
Other kidney disease	17%	9%	24%	
Months on dialysis	11 (10)	12 (11)	10 (9)	0.48
Dialysis access (fistula)	77%	83%	72%	0.38
Baseline CMR parameters				
LVEF	51 (9)	50 (10)	53 (7)	0.39
LV CI	3.3 (0.8)	3.3 (0.9)	3.3 (0.6)	0.75
LV GLS	− 21 (5)	− 21 (5)	− 22 (4)	0.86
LV GCS	− 33 (8)	− 32 (8)	− 33 (7)	0.39
LV GRS	− 22 (6)	− 21 (7)	− 23 (4)	0.45
RV GLS	− 24 (6)	− 23 (6)	− 24 (6)	0.56
LA EF	43 (14)	41 (14)	44 (13)	0.49
LA GLS	23 (13)	22 (16)	24 (11)	0.60
LA GCS	23 (11)	21 (10)	25 (11)	0.21
T1 time (ms)	1018 (40)	1023 (43)	1014 (40)	0.47
T2 time (ms)	50 (2.5)	50 (3.0)	50 (2.0)	0.92

*ALFA* alfacalcidol; *CI* cardiac index; *CMR* cardiac magnetic resonance imaging; *EDV* end-diastolic volume; *EF* ejection fraction; *ESRD* end-stage renal disease; *ETEL* Etelcalcetide; *GCS* global circumferential strain; *GLS* global longitudinal strain; *GRS*: global radial strain; *LA*: left atrial; *LV*: left ventricle; *RV*: right ventricle

### Change of natriuretic peptide levels

**Fig. 3:**
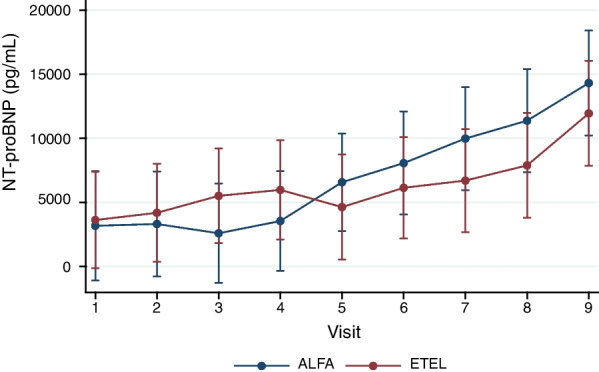
Descriptive overview of longitudinal NT-proBNP levels under ALFA and ETEL treatment. Data are summarized through predicted values and the 95%CI at the visit displayed. *ALFA* Alfacalcidol; *ETEL* Etelcalcetide; *NT-proBNP* N-terminal pro hormone brain natriuretic peptide

Figure 3 displays the change of NT-proBNP over time, stratified by treatment group. We did not observe a difference in natriuretic peptide levels (p = 0.294, ANCOVA adjusted for baseline NT-proBNP, study site, and residual renal function).

### Change of conventional and advanced CMR parameters

**Fig. 4:**
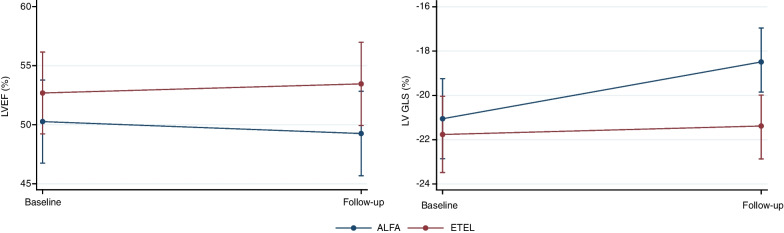
Descriptive overview of longitudinal LVEF and LV GLS values under ALFA and ETEL treatment. Data are summarized through predicted values and the 95%CI at the visit displayed. *ALFA* Alfacalcidol; *EF* ejection fraction; *ETEL* Etelcalcetide; *LV* left ventricle; *GLS* global longitudinal strain

At the initial CMR, patients presented with preserved LVEF (51 ± 9%) and longitudinal function as measured by LV GLS (− 21 ± 5%; see Fig. 4). Details on other conventional and advanced CMR parameters are listed in Table 1.

**Fig. 5:**
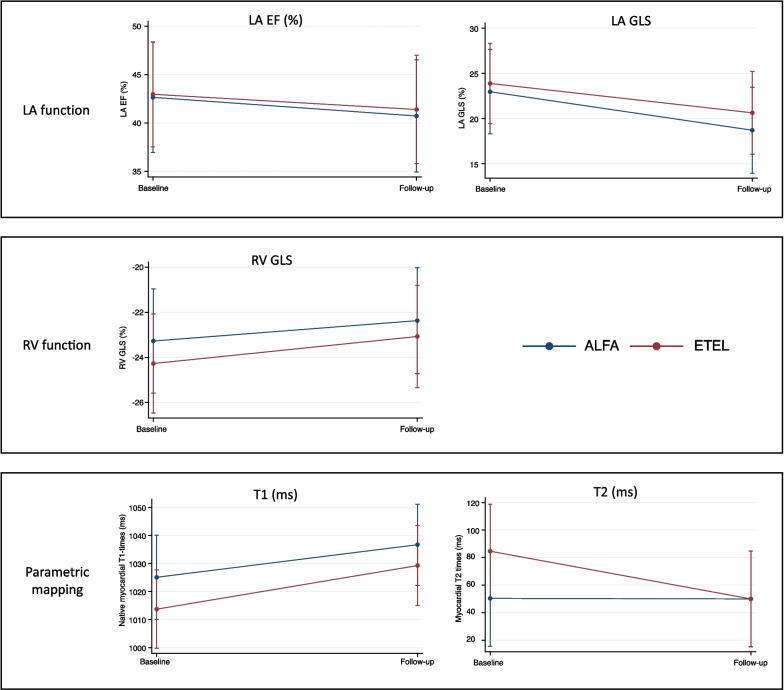
Descriptive overview of longitudinal CMR values under ALFA and ETEL treatment. Data are summarized through predicted values and the 95%CI at the visit displayed. *ALFA* Alfacalcidol; *EF* ejection fraction; *ETEL* Etelcalcetide; *GLS* global longitudinal strain; *LA* left atrium; *RV* right ventricle

The change in each CMR parameter is listed in Table 2 and Fig. 5. In addition, as previously shown, there was a protective effect against LV mass increase by ETEL, and patients receiving ETEL had a stable LV GLS over time. In contrast, patients randomized to ALFA showed deteriorated LV longitudinal function (+ 2.6 ± 4.6% compared to + 0.3 ± 3.8% in ETEL; per-protocol dataset). The key finding of protection of LV deterioration under ETEL treatment remained unchanged when analyzing the intention-to-treat dataset of patients with follow-up CMR, excluding the 4 patients with insufficient imaging quality (n = 55, Additional file 1: Table S2).

**Table 2:** Mean change of advanced CMR parameters during study period

Mean change in CMR parameter at follow-up CMR				
	Total	Alfa	Etl	p-value for treatment effect *
	N = 48	N = 23	N = 25	
LVEF	0.2 (7.8)	− 0.4 (9.5)	0.8 (6.0)	0.513
LV CI	0.19 (0.82)	0.27 (0.98)	0.11 (0.65)	0.998
LV GLS	1.4 (4.3)	2.6 (4.6)	0.3 (3.8)	0.045
LV GCS	0.6 (8.1)	0.4 (9.1)	0.8 (7.3)	0.367
LV GRS	− 0.4 (5.7)	− 0.1 (5.7)	− 0.8 (5.7)	0.381
RV GLS	1.0 (6.1)	0.8 (6.4)	1.1 (5.9)	0.982
LA EF	− 2.4 (9.7)	− 1.6 (8.6)	− 3.0 (10.6)	0.684
LA GLS	− 4.2 (9.4)	− 4.5 (10.9)	− 4.0 (7.9)	0.390
T1 time (ms)	12 (46)	15 (42)	10 (50)	0.411
T2 time (ms)	− 0.42 (2.43)	− 0.13(2.45)	− 0.70 (2.43)	0.652

Change of cardiovascular magnetic resonance imaging (CMR) parameters are displayed as mean (SD) difference from baseline CMR to follow-up CMR. Per-protocol dataset excluding patients with insufficient image quality

*CI* cardiac index; *EDV* end-diastolic volume; *EF* ejection fraction; *GCS* global circumferential strain; *GLS* global longitudinal strain; *GRS* global radial strain; *LA* left atrial; *LV* left ventricle

^*^p-value from repeated measure ANCOVA adjusting for baseline CMR parameter, study site, and residual renal function

We observed no differences in other conventional or advanced CMR parameters (Table 2). The adjusted mean change of LV GLS estimated by the ANCOVA was − 1.1% (95% CI, − 2.3 to − 0.0%, p = 0.045) when comparing the ETL to the ALFA group, and adjusted for baseline LV GLS, study site, and residual renal function.

In an exploratory step, we found that drug treatment remained significantly associated with a mean change in LV GLS when adjusted for LV mass index change and an FGF23 change (Table 3). The trajectories of PTH over time were similar between treatment groups and the change of calcium, phosphate and PTH had no impact on the change of LV GLS.

**Table 3:** Association between mean change LV GLS and baseline variables

		95%CI		p-value
	Coefficient	LCI	UCI	
Univariate analysis				
Age	− 0.02	− 0.14	0.10	0.750
Male sex	3.29	0.53	6.06	0.020
Diabetes	1.53	− 0.98	4.03	0.226
Hypertension	2.00	− 6.87	10.86	0.652
Months on dialysis	0.003	− 0.122	0.128	0.960
Etelcalcetide (ETL) treatment	− 2.69	− 5.12	− 0.26	0.039
Multivariate analysis				
Etelcalcetide (ETL) treatment	− 2.62	− 5.10	− 0.14	0.042

Regression models demonstrating the association between mean change of LV GLS from baseline variables. The multivariate model includes baseline LV GLS sex, LV mass change, and FGF23 change; per-protocol dataset

FGF23: Fibroblast growth factor 23, GLS: global longitudinal strain, LV: left ventricle

T2 relaxation times increased as a function of dialysis duration in both treatment groups (Additional file 1: Table S1, Figure S1).

## Discussion

In this trial, we demonstrated that ETEL, in comparison to ALFA treatment, prevented the decline of LV longitudinal function, as evaluated using FT-CMR, in hemodialysis patients over a one-year period. Our findings revealed no difference in myocardial tissue characteristics, as observed through T1- and T2-mapping, between the treatment groups.

There was no significant difference in LVEF between the groups at one-year follow up. Despite the high prevalence of cardiovascular events and progressive symptoms of HF, the majority of patients with CKD exhibit preserved EF [30–32]. Although reduced LVEF is a significant prognostic indicator, it typically occurs in the later stages of uremic cardiomyopathy development, and less than 20% of ESRD patients exhibit detectable systolic dysfunction [33]. Furthermore, LVEF is influenced by changes in loading conditions, which can vary dramatically during interdialytic intervals. It has been demonstrated that GLS enhances the ability to detect early subclinical LV systolic dysfunction in this patient population [32, 34–36]. Although HFrEF is observed in only a minority of patients undergoing hemodialysis, diastolic dysfunction and HF with preserved EF are significantly prevalent within this patient collective [7]. Among these patients impaired LV GLS was found to be prevalent, suggesting the existence of concealed systolic dysfunction despite having a normal LVEF [5]. In patients with intermediate to terminal stages of CKD, GLS has been demonstrated to be a more sensitive and independent predictor of both overall and cardiovascular mortality when compared to EF. Therefore, GLS provides additional prognostic value in patients with preserved EF [8, 34, 37]. CMR is widely regarded as the established reference standard for the assessment of cardiac mass and volume [8, 38]. Our findings may underscore the potential suitability of CMR for early detection of subclinical HF and monitoring treatment progress, particularly in this specific patient population, in contrast to relying solely on EF. Barbosa et al. conducted a trial examining patients who underwent kidney transplantation, wherein LV GLS was assessed using FT-CMR. The results revealed improvements in GLS after six months, whereas there was no significant increase in LVEF during the follow-up period [39].

It is worth noting that our patients initially presented with mean LV GLS and EF values that fell within the normal range [40, 41]. Patients with significantly impaired LV systolic function were excluded from the trial due to their substantially higher mortality rates [9, 22]. Furthermore, our patient population had been undergoing dialysis for a relatively brief duration at the beginning of the trial (11 months on average). LV structural and function abnormalities, which contribute to cardiovascular disease and an elevated risk of mortality, are not only highly prevalent in patients with ESKD, but they also tend to progress over time during dialysis treatment [42, 43]. Hence, it is reasonable to expect a gradual decline in LV systolic function over time among patients undergoing dialysis. Previous findings have indicated that LVH serves as a compensatory mechanism to offset impaired LV GLS and maintain LVEF. Consequently, improvements in LV GLS should contribute to a reduction in LVH, and vice versa, while LVEF remains unchanged [44]. Notably, in our trial, the protective effect of ETL against the decline of LV longitudinal function was not linked to the previously observed changes in LV mass or FGF23. This suggests that the mechanism responsible for the treatment-induced inhibition of LVH may differ from the one underlying the preservation of LV longitudinal function.

The translation of the observed prevention of LV longitudinal function decline into clinical outcomes remains uncertain. The EVOLVE study, the largest trial investigating the impact of calcimimetic treatment in hemodialysis patients, did not demonstrate a significant difference in the primary composite outcome of time until death or the occurrence of the first nonfatal cardiovascular event [45]. The interpretation of this trial is considerably constrained due to a substantial number of dropouts in the treatment group (62%) and a high rate of crossover in the placebo group (20%) [46]. In contrast to the EVOLVE study, the medication in our trial was administered intravenously, enabling a dependable and consistent delivery of the study drug. This aspect is crucial, particularly considering the significant rate of non-adherence observed for calcimimetics in this patient population [47]. It is essential to consider that the follow-up period in our trial was limited to 12 months, and the study participants had been undergoing dialysis for less than a year at the commencement of the trial. Typically, patients with ESKD require dialysis treatment for several years. Nevertheless, as our study did not explore the impact of the medication on definitive endpoints, given its relatively small size, we can only speculate on whether the observed deterioration could potentially hold clinical significance over time [42, 43].

Although previous studies have demonstrated a correlation between impaired LV GLS and elevated plasma levels of NT-proBNP, our study observed a similar increase in both treatment groups [48]. This finding can be attributed to the consistent monitoring of stable loading conditions in the closely monitored patient cohort throughout the trial [9, 49, 50].

Individuals with kidney disease exhibit a notable presence of interstitial myocardial fibrosis, which intensifies with the duration of dialysis treatment. This fibrosis is associated with cardiac hypertrophy, resulting in ventricular stiffness and subsequent impairment of both systolic and diastolic function. Additionally, it contributes to a heightened risk of arrhythmias [51, 52]. In CKD, the interstitial fibrosis is diffuse, not restricted to the coronary region, and advances gradually over time, eventually leading to replacement fibrosis. Even years after kidney transplantation, this fibrosis does not completely reverse [51]. Our study revealed no differences in T1-mapping, which is a surrogate of myocardial fibrosis, from baseline to one year of treatment, between study medications. It is crucial to note that our study cohort exhibited T1 values at the upper limit of normal at baseline, indicating that they did not yet demonstrate indications of advanced fibrosis in parametric mapping [53, 54]. As previously mentioned, the likely reason for this finding can be attributed to the relatively short duration of dialysis treatment at the beginning of the study. In addition, the T1 values could also have been driven by the slightly increased water content of myocardial tissue, as reported in T2 relaxation times [20, 55]. The development of cardiac fibrosis varies depending on the underlying causes, ranging from weeks following myocardial injury caused by infarction to years in cases of diabetic cardiomyopathy or hypertensive heart disease [56]. Hence, it is important to consider that visualizing tissue alterations after the initiation of treatment, particularly when employing native CMR techniques like T1 mapping, may require a significantly longer period than 12 months. This finding aligns with the previously mentioned trial in patients following kidney transplantation, which did not demonstrate significant correlations between changes in LV GLS and native T2 or T2 measurements [39]. While there have been suggestions of an association between strain parameters and the severity of interstitial myocardial fibrosis and hypertrophy in uremia, it has also been demonstrated that the correlation between them is minimal. This suggests that they may reflect different domains of myocardial disease [37, 57]. Although no differences were observed in T2-mapping between the treatment groups, it was noted that T2 values were associated with the length of time on dialysis. This association could potentially be attributed to a more effectively controlled fluid volume status.

### Limitations

One significant limitation of this trial was the small sample size, which was initially determined to detect an effect of the study medication on a different parameter, specifically LV mass. Additional limitations of this study include the relatively short duration of follow-up, which may not capture long-term changes in myocardial remodeling that develop over the course of years, as well as the absence of data on hard cardiovascular outcomes. In line with the EVOLVE study protocol, patients with significantly impaired LV systolic function were excluded from the trial, which represents an additional limitation in this analysis [45]. Moreover, due to the study setting involving patients with ESKD, the use of contrast agents was not feasible, resulting in the utilization of native T1 relaxation time instead. As a result, the assessment of extracellular expansion through post-contrast T1 mapping could not be included in the analysis. As this trial did not examine hard endpoints, it is not possible to make definitive statements regarding the clinical relevance of the findings.

One of the major strengths of our trial was its prospective design with randomization, ensuring a robust and reliable study methodology. As described in our previous publication, thorough assessments of blood pressure and fluid volume status were conducted to minimize potential confounding factors related to volume and pressure overload on myocardial remodeling and function [9]. Although strain imaging by echocardiography is more readily available compared to CMR, it is accompanied by certain limitations, such as restricted acoustic windows, potential image quality issues, and variability in interpretation both between observers and within the same observer. Furthermore, the occurrence of fluid shifts associated with hemodialysis can potentially compromise the accuracy and reliability of this imaging technique [35].

## Conclusions

In summary, our findings indicate a trend towards the preservation of LV longitudinal function with ETEL medication compared to ALFA in hemodialysis patients, although no significant difference was observed in LVEF. These findings suggest that LV GLS may be a more sensitive parameter for the diagnosis of LV dysfunction and for monitoring treatment in this patient population, providing additional support for its potential clinical utility. Given that this analysis was a secondary investigation, the hypothesis regarding LV longitudinal function necessitates further validation in a larger cohort.

### Supplementary Information

**Additional file 1: Table S1.** Association between length of dialysis and baseline CMR parameters. **Table S2.** Mean change of advanced CMR parameters during study period. Intention-to-treat dataset with follow-up CMR. **Figure S1.** T2-mapping.

## Data Availability

The datasets used during the current study are available from the corresponding author on reasonable request.

## Abbreviations

ALFA: Alfacalcidol
CKD: Chronic kidney disease
CMR: Cardiac magnetic resonance imaging
EF: Ejection fraction
ESKD: End-stage kidney disease
ETEL: Etelcalcetide
EVOLVE: Evaluation of Cinacalcet Hydrochloride Therapy to Lower Cardiovascular Events
FGF23: Fibroblast growth factor 23
FT: Feature-tracking
GLS: Global longitudinal strain
LV: Left ventricle
LVH: Left ventricular hypertrophy
NT-proBNP: N-terminal pro hormone brain natriuretic peptide
PTH: Parathyroid hormone
