# Supplementary material for: Effect of etelcalcetide versus alfacalcidol on left ventricular function and feature-tracking cardiac magnetic resonance imaging in hemodialysis—a post-hoc analysis of a randomized, controlled trial
Source: J Cardiovasc Magn Reson. 2023 Nov 6;25:62. doi: 10.1186/s12968-023-00975-4 (PMC10626812; doi:10.1186/s12968-023-00975-4)
Supplement: Supplementary file 1 — Additional file 1: Table S1. Association between length of dialysis and baseline CMR parameters. Table S2. Mean change of advanced CMR parameters during study period. Intention-to-treat dataset with follow-up CMR. Figure S1. T2-mapping. [file 12968_2023_975_MOESM1_ESM.docx]

Etelcalcetide inhibits deterioration of left ventricular function on feature-tracking cardiac magnetic resonance imaging in hemodialysis

**Additional data**

Table S1: Association between length of dialysis and baseline CMR parameters

| Parameter | Adj. Coef. | LCI | UCI | p-value |
| --- | --- | --- | --- | --- |
| T1 | -0.672 | -1.693 | 0.349 | 0.193 |
| T2 | -0.071 | -0.14 | -0.002 | 0.044 |
| LVEDV/BSA | 0.191 | -0.403 | 0.785 | 0.522 |
| LVEF | -0.097 | -0.337 | 0.143 | 0.420 |
| LV CI | 0.02 | -0.002 | 0.041 | 0.069 |
| LV GLS | 0.117 | -0.005 | 0.239 | 0.059 |
| LV GCS | 0.043 | -0.149 | 0.234 | 0.655 |
| LV GRS | 0.211 | 0.075 | 0.346 | 0.003 |
| LA GLS | -0.29 | -0.638 | 0.058 | 0.101 |
| RV GLS | 0.096 | -0.057 | 0.248 | 0.214 |

BSA: body surface area; EDV: end-diastolic volume; EF: ejection fraction; GCS: global circumferential strain; GLS: global longitudinal strain; GRS: global radial strain; LA: left atrial; LV: left ventricle

Table S2: Mean change of advanced CMR parameters during study period. Intention-to-treat dataset with follow-up CMR

|  | Mean change in CMR parameter  at follow-up CMR | | |  | |  |
| --- | --- | --- | --- | --- | --- | --- |
|  | Total | Alfa | Etel | | p-value for treatment effect * | |
|  | N=55 | N=26 | N=28 | |  | |
| LVEF | -2.0 (9.4) | -1.3 (8.6) | -2.5 (10.2) | | 0.64 | |
| LV CI | 0.15 (0.81) | 0.21 (0.98) | 0.10 (0.62) | | 0.64 | |
| LV GLS | 1.5 (4.2) | 2.7 (4.5) | 0.4 (3.7) | | 0.040 | |
| LV GCS | 0.3 (7.9) | 0.5 (8.7) | 0.0 (7.3) | | 0.81 | |
| LV GRS | -0.7 (5.6) | -0.4 (5.6) | -1.0 (5.7) | | 0.66 | |
| RV GLS | 1.2 (6.1) | 0.9 (6.7) | 1.4 (5.7) | | 0.78 | |
| LA EF | -2.0 (9.4) | -1.3 (8.6) | -2.5 (10.2) | | 0.64 | |
| LA GLS | -4.0 (9.1) | -4.1 (10.7) | -4.0 (7.6) | | 0.98 | |
| T1 time (ms) | 14 (47) | 14 (44) | 15 (50) | | 0.98 | |
| T2 time (ms) | -0.32 (2.40) | -0.17 (2.33) | -0.47 (2.49) | | 0.66 | |

Change of cardiovascular magnetic resonance imaging (CMR) parameters are displayed as mean (SD) difference from baseline CMR to follow-up CMR.

CI: cardiac index; EDV: end-diastolic volume; EF: ejection fraction; GCS: global circumferential strain; GLS: global longitudinal strain; GRS: global radial strain; LA: left atrial; LV: left ventricle

* p-value from repeated measure ANCOVA adjusting for baseline CMR parameter, study site, and residual renal function.

Figure S1: T2-mapping

Dialysis treatment time and T2 relaxation times of the entire study cohort
